# Adaptive interventions for optimizing malaria control: an implementation study protocol for a block-cluster randomized, sequential multiple assignment trial

**DOI:** 10.1186/s13063-020-04573-y

**Published:** 2020-07-20

**Authors:** Guofa Zhou, Ming-chieh Lee, Harrysone E. Atieli, John I. Githure, Andrew K. Githeko, James W. Kazura, Guiyun Yan

**Affiliations:** 1 Program in Public Health, University of California, Irvine, CA USA; 2 Department of Public Health, Maseno University, Kisumu, Kenya; 3 Kenya Medical Research Institute, Kisumu, Kenya; 4 Center for Global Health and Diseases, Case Western Reserve University, Cleveland, OH USA

**Keywords:** Adaptive intervention, Sequential multiple assignment randomized trial, Block-cluster randomized, Long-lasting insecticidal net (LLIN), Indoor residual spraying, Piperonyl butoxide-treated LLIN, Larval source management, Clinical malaria incidence rate, Active case surveillance, Cost-effectiveness, *Q*-learning

## Abstract

**Background:**

In the past two decades, the massive scale-up of long-lasting insecticidal nets (LLINs) and indoor residual spraying (IRS) has led to significant reductions in malaria mortality and morbidity. Nonetheless, the malaria burden remains high, and a dozen countries in Africa show a trend of increasing malaria incidence over the past several years. This underscores the need to improve the effectiveness of interventions by optimizing first-line intervention tools and integrating newly approved products into control programs. Because transmission settings and vector ecologies vary from place to place, malaria interventions should be adapted and readapted over time in response to evolving malaria risks. An adaptive approach based on local malaria epidemiology and vector ecology may lead to significant reductions in malaria incidence and transmission risk.

**Methods/design:**

This study will use a longitudinal block-cluster sequential multiple assignment randomized trial (SMART) design with longitudinal outcome measures for a period of 3 years to develop an adaptive intervention for malaria control in western Kenya, the first adaptive trial for malaria control. The primary outcome is clinical malaria incidence rate. This will be a two-stage trial with 36 clusters for the initial trial. At the beginning of stage 1, all clusters will be randomized with equal probability to either LLIN, piperonyl butoxide-treated LLIN (PBO Nets), or LLIN + IRS by block randomization based on their respective malaria risks. Intervention effectiveness will be evaluated with 12 months of follow-up monitoring. At the end of the 12-month follow-up, clusters will be assessed for “response” versus “non-response” to PBO Nets or LLIN + IRS based on the change in clinical malaria incidence rate and a pre-defined threshold value of cost-effectiveness set by the Ministry of Health. At the beginning of stage 2, if an intervention was effective in stage 1, then the intervention will be continued. Non-responders to stage 1 PBO Net treatment will be randomized equally to either PBO Nets + LSM (larval source management) or an intervention determined by an enhanced reinforcement learning method. Similarly, non-responders to stage 1 LLIN + IRS treatment will be randomized equally to either LLIN + IRS + LSM or PBO Nets + IRS. There will be an 18-month evaluation follow-up period for stage 2 interventions. We will monitor indoor and outdoor vector abundance using light traps. Clinical malaria will be monitored through active case surveillance. Cost-effectiveness of the interventions will be assessed using *Q*-learning.

**Discussion:**

This novel adaptive intervention strategy will optimize existing malaria vector control tools while allowing for the integration of new control products and approaches in the future to find the most cost-effective malaria control strategies in different settings. Given the urgent global need for optimization of malaria control tools, this study can have far-reaching implications for malaria control and elimination.

**Trial registration:**

US National Institutes of Health, study ID NCT04182126. Registered on 26 November 2019.

## Background

Tremendous progress has been made in malaria control in Africa over the past two decades. This is the result of effective vector interventions, particularly the massive scale-up of long-lasting insecticide-treated nets (LLINs) and indoor residual spraying (IRS), as well as improvements in diagnostic testing and expanded availability of artemisinin-combination therapy (ACT). Nonetheless, current first-line interventions are not sufficient to eliminate malaria in many countries, and a dozen countries in Africa show a trend of increasing malaria incidence over the past several years [1]. Vector control is an important component of national malaria control strategies in Africa, and the core vector control methods are LLINs, IRS, and larval source management (LSM) [2, 3]. However, the massive scale-up of LLINs and IRS has led to major changes in vector biology, which pose significant new challenges to malaria control and elimination.

Insecticide resistance is rising rapidly, and pyrethroid resistance has been documented in malaria vectors in most countries throughout the Afrotropical region [4]. All major malaria vectors in Africa, including *Anopheles gambiae*, *Anopheles funestus*, and *Anopheles arabiensis*, are highly resistant to pyrethroids [5–7] and to multiple classes of insecticides [8, 9], resulting in limited viable insecticides for IRS. In addition to the significant problem posed by insecticide resistance, outdoor transmission is becoming increasingly common. Recent studies document a behavioral shift in malaria vectors, from midnight biting to biting in the early evening and morning when people are outdoors and not protected by IRS or LLINs [10–12]. The first-line control measures (LLINs and IRS) only protect residents sleeping under nets or resting indoors. Outdoor malaria transmission has become a very important challenge to malaria control. More importantly, malaria risk is dynamic and spatially heterogeneous. Malaria risk fluctuates over time and is associated with the success of control programs [13–16]. It may vary among villages due to micro-geographic variations in vector ecology [17, 18], residents’ health-seeking behaviors [19], socioeconomic factors, and other reasons [20–22]. The effectiveness of a control method in one setting is not always guaranteed elsewhere. Control methods need to be adapted to local malaria risks and vector ecology [23].

The problem of how to optimize intervention strategies to maintain the current progress toward eventual malaria elimination has become an important one. In Africa, the core malaria vector control tools are LLINs, IRS, and LSM [2, 3]. However, there is little knowledge regarding how these interventions should be combined in order to optimize their impact on the malaria burden. IRS is an expensive method of malaria control [24], and LSM is labor intensive and requires strong and sustained community participation [25]. Under what epidemiological settings will IRS exhibit the highest impact, and at what frequency should it be applied? Where should LSM be implemented to further reduce malaria transmission and morbidity? In addition, it is imperative that newly approved control tools, e.g., next-generation nets, new larvicide formulations, and new classes of IRS insecticides, be incorporated into control programs. How, when, and where should these tools be integrated into malaria control programs that rely on current first-line intervention tools?

In recent field tests, some new intervention tools have shown promise against insecticide-resistant malaria vectors and outdoor transmission. These include long-lasting piperonyl butoxide-treated nets (PBO Nets), long-lasting larvicides, and non-pyrethroid insecticides for IRS. All currently deployed LLINs worldwide are pyrethroid-based, despite high insecticide resistance in malaria mosquito vectors [26]. Recently, next-generation LLINs combining the synergist PBO with pyrethroids were recommended by the WHO to combat pyrethroid resistance [27]. PBO enhances the effects of pyrethroids on mosquito vectors, thus reducing vector resistance. Field studies in Africa found that compared to regular LLINs, PBO Nets significantly reduced malaria transmission in areas of high pyrethroid resistance [28–33]. Larval source management has been recommended as a public health intervention tool in specific locations where habitats are relatively few and readily identified [2, 3]. New US Environmental Protection Agency (EPA)-approved long-lasting microbial larvicides are now available. These larvicides are released slowly, thereby increasing their effective duration to 3–5 months and reducing operational costs [34]. On the other hand, the dynamic and cryptic nature of larval habitats may allow for only a portion of habitats to be identified and treated. Would new long-lasting larvicides, potentially more cost-effective than larvicides used in the past, provide added benefit to malaria control? Apart from pyrethroid insecticide IRS, new classes of insecticides, e.g., Actellic, an organophosphate, have recently been introduced for IRS in Africa [2, 3]. Field tests in several African countries found that these new IRS treatments significantly reduced malaria transmission in areas with moderate to high vector resistance to pyrethroids [15, 28, 35]. These non-pyrethroid insecticides are expected to be more expensive but also more effective against pyrethroid-resistant vectors. Given the prevalent outdoor resting behavior of mosquito vectors, will the non-pyrethroid insecticides provide added benefit and be cost-effective for malaria control?

The commonly used trial design in malaria control is randomized controlled trials (RCTs) or cluster-randomized trial (CRT) [28, 34, 36–39], which is considered the gold standard for assessing the relative efficacy of competing treatment options in evidence-based disease management [40]. Future malaria control strategies will likely involve in combination of different types of interventions, such as LLINs plus LSM or other interventions to control insecticide-resistant and outdoor vectors [28, 34, 36–39, 41–45]. Due to the potential large sample size requirement, a complete factorial design of RCT or CRT is not a practical way to find the robust combination among the many available interventions. A multi-stage adaptive intervention may be an appropriate and cost-saving approach. An adaptive design is loosely defined as a trial design that allows modifications to the trial procedure after its initiation without undermining its validity and integrity [46–49]. One way to inform the development of adaptive intervention is to randomly sequentially (during different stages of the intervention) assign different interventions to different arms or different subjects to different interventions, i.e., the sequential multiple assignment randomized trial (SMART) [46]. Unlike in conventional RCT/CRT, which treats all interventions/subjects equally and fixed throughout a trial, in adaptive interventions, decisions must be made concerning if and when an intervention needs to be continued or replaced or terminated, and accordingly, which intervention should follow. In this context, future subjects are randomized with bias toward the best-performing interventions. Adaptive design has frequently been used in clinical studies in areas such as psychology, mental health, and cancer treatment [46–49], but it has not been used in studies on vector and vector-borne infectious disease control.

The aim of this trial is to design optimal adaptive combinations of vector control interventions to maximize reductions in malaria burden based on local malaria transmission risks, vector ecology, and the available mix of interventions approved by the Ministry of Health (MoH) of Kenya. The hypothesis is that an adaptive approach based on local malaria risk and changing vector ecology will lead to significant reductions in malaria incidence and transmission risk. This paper describes a protocol for finding the optimal combination of interventions using a cluster-randomized sequential multiple assignment randomized trial (SMART) design in Kenya.

## Methods/design

### Hypothesis, interventions, and endpoint outcomes

#### Hypothesis

An adaptive approach based on local malaria risk and changing vector ecology will lead to significant reductions in malaria incidence and transmission risk.

#### Objective

The central objective of this trial is to design optimal adaptive combinations of vector control interventions to maximize reductions in malaria burden based on local malaria transmission risks, vector ecology, and the available mix of interventions approved by the Ministry of Health of Kenya.

#### Trial design

**Fig. 1:**
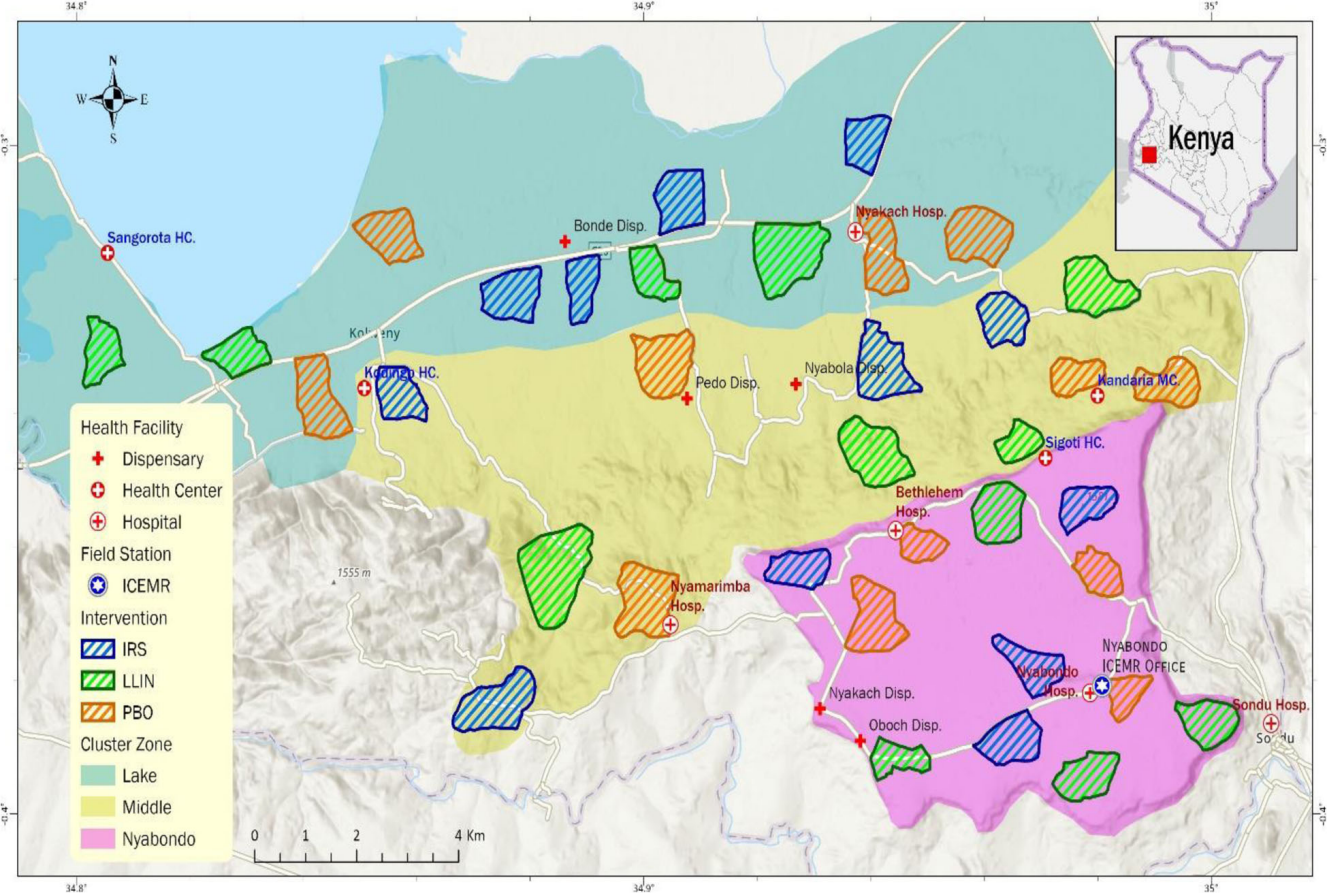
Study site; distribution of trial clusters and initial interventions. Block zones are shown in different background colors; initial intervention in each cluster is shown in different boundary colors. LLIN, long-lasting insecticidal net; PBO, piperonyl butoxide-treated LLIN; IRS, indoor residual spraying

This is an open-label, block-cluster randomized, controlled, sequential multiple assignment trial with a variable number of arms (adaptive design) and a baseline period without crossover. A potential trial design is shown in Fig. 1 and Table 1.

**Table 1:**
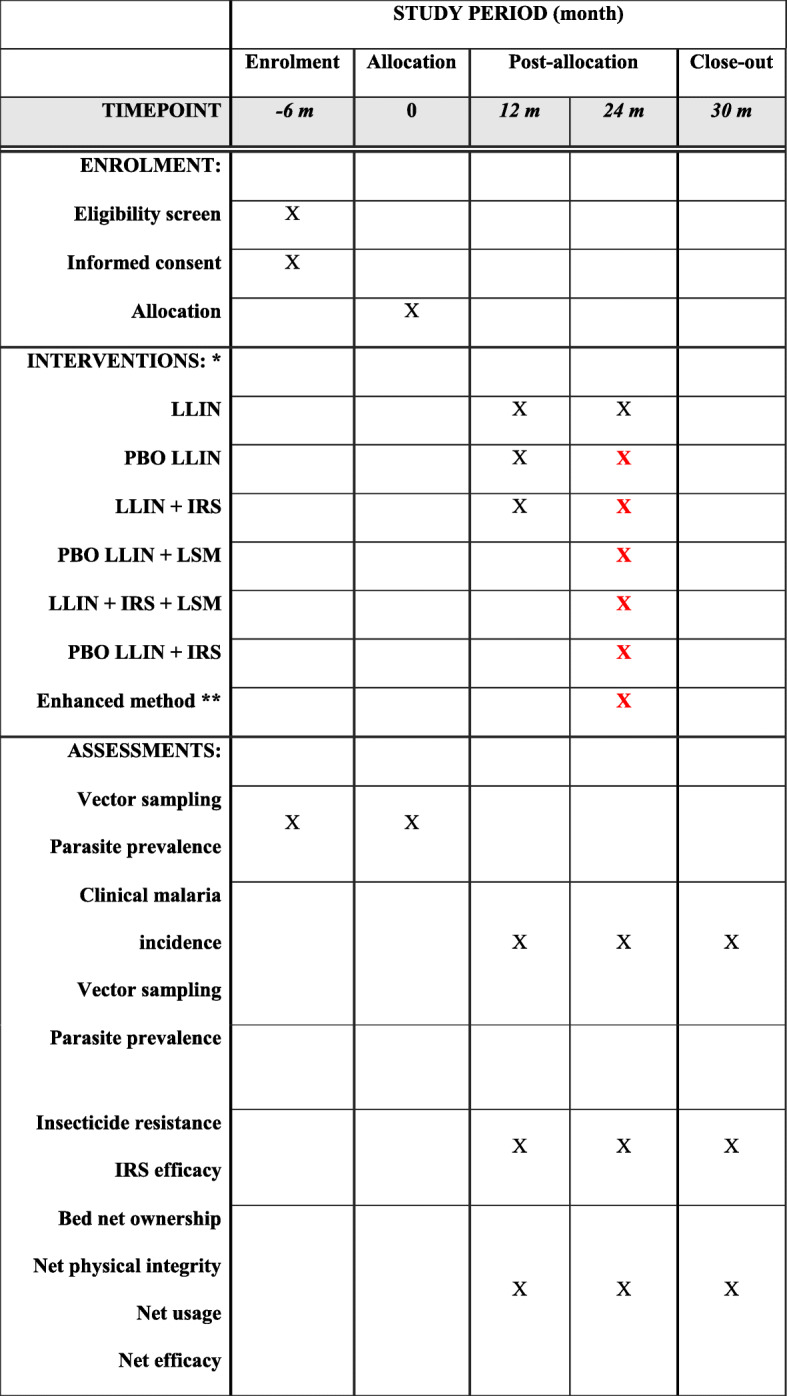
Content and timelines for the schedule of enrolment, interventions, and assessments

*Due to the adaptive nature of the design, the initial interventions (year 1) are fixed; however, the subsequent interventions depend on the outcomes from initial interventions, i.e., effective interventions will be continued; otherwise, other interventions will be introduced. Black color represents definite interventions, and red color represents all possible interventions depend on outcomes from previous stage of interventions. *LLIN* long-lasting insecticidal net, *PBO LLIN* piperonyl butoxide-treated LLIN, *IRS* indoor residual spraying, *LSM* larval source management

**Enhanced method: the most cost-effective method determined by machine learning based on outcomes from previous interventions

#### Interventions

##### LLIN

We will use the currently implemented LLINs, which are the standard intervention administrated by the MoH. These include Olyset LLIN (Sumitomo Chemical UK PLC, London, UK), which contains 2% permethrin with 150 denier yarn, and PermaNet 2.0 (Vestergaard, Lausanne, Switzerland) containing 1.8 g/kg (75 denier yarn) or 1.4 g/kg (100 denier yarn) of deltamethrin. For LLIN clusters, no additional nets will be supplied, since this intervention is the government-administered standard intervention and current coverage is very high (95%) in our study area. In addition, local government-run hospitals, clinics, and health centers routinely distribute nets to pregnant women and children under 5 years old, and therefore, the coverage is well maintained.

##### PBO LLIN

We will test the Olyset Plus LLIN (Sumitomo Chemical UK PLC, London, UK), which contains 2% permethrin and 1% PBO. For the PBO Net clusters, each household will be provided one Olyset Plus per two people along with appropriate education, which is the same as the standard LLIN allocation practice [2, 3]. Residents will be asked to use the PBO Nets provided, and net usage will be monitored.

##### Actellic® IRS

For the IRS, we will use the micro-encapsulated pirimiphos-methyl, Actellic 300CS (Syngenta Crop Protection AG, Basel, Switzerland), with an AI concentration of 300 g/L. Actellic 300CS IRS has been implemented in several African countries [35, 50–52]. For IRS clusters, each dwelling’s interior walls and ceiling will be sprayed with the recommended dosage of 1 g/m^2^ at the recommended frequency of once a year. This is also the current PMI-implemented spraying frequency in several African countries [50–52].

##### LSM

LSM will be implemented in selected clusters and will include the physical filling or removal of temporary larval habitats and the larviciding of semi-permanent and permanent habitats, per Kenya’s National Malaria Strategic Plan [2, 3]. For the larviciding, we will use the long-lasting microbial larvicide manufactured by Central Life Sciences (Sag Harbor, NY, USA) with active ingredients *Bacillus thuringiensis israelensis* (Bti) (6% by weight) and *Bacillus sphaerius* (Bs) (1% by weight). This product has been proven effective against insecticide-resistant mosquitoes [34]. Application dosage will follow the recommendation of the manufacturer: semi-permanent and permanent habitats will be treated with FourStar® 180-day Briquets with one briquet per 100 ft^2^ of water surface, regardless of water depth. Re-treatment will occur every 4 to 5 months.

#### Primary and secondary endpoints

The primary endpoint is clinical malaria incidence rate. The secondary endpoints are malaria vector abundance and transmission intensity. A clinical malaria case is defined as an individual with fever (axillary temperature of 37.5 °C or higher) and other related symptoms such as chills, severe malaise, headache, or vomiting, in the presence of a *Plasmodium*-positive blood smear. Clinical malaria incidence rate is calculated as the number of clinical malaria episodes divided by the total person-time (person-years) at risk based on demographic surveys. Malaria vector abundance is measured as the total density of malaria vector mosquitoes (*An. gambiae* s.s., *An. arabiensis*, *An. funestus*, and other new species capable of transmitting malaria) collected indoors by CDC miniature light traps. Malaria transmission intensity is measured as the sum of the indoor entomological inoculation rates (EIRs).

### Study area and cluster selection

We will conduct our study initially in 36 randomly selected clusters in an area consisting of both low- and high-elevation localities (1100 m to 1700 m altitude) in the Lake Victoria shore area southeast of Kisumu County, western Kenya (34° 49′ E to 34° 59′ E, 0° 15′ S to 0° 22′ S) (Fig. 1). A cluster is a village or several neighboring villages, typically covering an area of approximately 2 km^2^ and comprising 200–300 households and about 500–1000 inhabitants. A village is the smallest administrative unit in Kenya. For simplicity of management and field surveys, cluster boundaries will coincide with administrative boundaries. These boundaries have been mapped using ArcGIS prior to any field surveys based on Kisumu County administrative maps. Clusters are selected at about 2 km apart to avoid any spill-over effect. The catchment population of the study area, including all study clusters and buffer zones, is estimated as 100,000 according to 2010 census data.

Local residents are predominantly farmers who depend on crop farming and cattle and goat herding for subsistence. Malaria transmission is seasonal, with two peaks in vector abundance reflecting the bimodal rainfall pattern: a major peak between April and June and a minor peak between October and November [53]. Malaria is predominantly caused by *Plasmodium falciparum* [54]. The main malaria vectors in the area are *An. gambiae* s.s., *An. arabiensis*, and *An. funestus* s.l. [55]. In the trial study area, a 2017–2018 survey found that *An. funestus* accounted for 45% of all *Anopheles* captured, followed by *An. gambiae* s.l. (37%), and other *Anopheles* species (18%), and 89% of *An. gambiae* s.l. are *An. arabiensis* [50]. In addition, outdoor transmission is high [50, 55]. The mosquito resistance to different insecticides which include pyrethroids has been reported in the study area and in the nearby Ahero area (10 km north of the trial site) [8, 50]. Although metabolic-based resistance has not been determined in the study area, the fact that *An. funestus* is the dominant malaria vectors in the area justifies the usefulness of PBO LLINs, because pyrethroid resistance in *An. funestus* is due mainly to metabolic-based resistance [56].

### Baseline surveys for cluster randomization

We will conduct entomological, epidemiological, and demographic surveys before the start of the interventions (Table 1). Together with other topographic and landscape variables, these surveys will provide baseline data for cluster stratification and randomized allocation of different interventions. The baseline data will also be used in evaluating the interventions.

#### Demographic and socioeconomic data

A baseline census of the population with house locations (mapped by spatial coordinates) will be conducted at the start of the study and updated annually. Demographic surveys will include all residents who live inside the study clusters. Each household and its family members will have a unique ID that will be used to trace the individuals’ visits to health centers for cross-sectional blood sampling and active malaria case surveillance. We will use our pre-established demographic surveillance system to track the population and its changes on an annual basis.

Data on socioeconomic status and malaria intervention practices will be obtained during the demographic surveillance, which will administer a separate questionnaire to all households in each study cluster. A series of questions will address socioeconomic status indicators (house size, ownership of electronics, land income, occupation, and others), LLIN ownership and usage, use of repellents, use of IRS, and malaria treatment-seeking behaviors.

#### Cross-sectional vector abundance and malaria infection prevalence

To determine malaria infection prevalence, cross-sectional finger-prick blood samples will be collected on blood films and on filter papers for PCR analysis, following the nested PCR method for dried blood spots based on the 18S rRNA gene to detect the malaria species [54, 57, 58]. In western Kenya, the major rainy season is usually from April to July and the major dry season is from November to March next year. To minimize the seasonality effect, we will conduct vector abundance and parasite prevalence surveys twice a year, once in June/July and another time from November to January.

All clusters will be examined for mosquito abundance. We have previously determined that the CDC light trap is an efficient sampling tool [55, 59]. For each cluster, we will conduct adult mosquito collections in 10 houses for 10 nights per month. *An. gambiae* s.l. and *An. funestus* s.l. will be analyzed by rDNA-PCR for species identity [60, 61]. All specimens will be tested for *Plasmodium* sporozoite infection using PCR [62, 63], and blood meal analysis will be performed by PCR [64–66]. Aquatic habitats within the 0.5-km buffer zone of the study cluster will be mapped using GPS and examined for *Anopheles* larval and pupal abundance using standard dippers [67]. A subset of larvae will be reared to adults and used to test resistance to pyrethroid and organophosphate insecticides using the standard WHO insecticide susceptibility bioassay [68]. *Plasmodium falciparum* isolates from the community-based and hospital-based surveillance will be genotyped at *Pfmdr*1 for lumefantrine tolerance and at *PfKelch*13 for artemisinin resistance [69].

#### Acquisition of other malaria risk predictor variables

A variety of predictor variables for malaria risk will be collected for each cluster: (1) human density and age structure; (2) topographic parameters, which are associated with mosquito larval habitat formation [70, 71]; (3) land-use and land-cover types related to the development and survival of Anopheline mosquito larvae and adults [72–74]; and (4) meteorological data. Topographic parameters such as elevation, slope, wetness index, flow distance to stream, aspect of land surface, and curvature will be obtained from the digital elevation model of the study site, which we have already developed. Land-use and land-cover data will be obtained from supervised classification of the most recent satellite images, and the normalized difference vegetation index (NDVI) will be computed. Meteorological data (temperature, rainfall, and vapor pressure) will be obtained from a global 0.5° × 0.5° gridded data set of monthly terrestrial surface climate from the World Meteorological Organization database.

### Cluster stratification for interventions

For each cluster, we will determine vector abundance and malaria infection prevalence. Malaria risk in each cluster will be predicted based on the predictive features (variables) described above. We plan to adopt two approaches for risk prediction. The first approach (classification-based) will first stratify the study clusters into distinct strata based on weighted vector abundance and malaria infection prevalence using *k*-means or hierarchical clustering algorithms [75]. A classifier will be trained to predict risk-group assignments based on predictive variables. To avoid overfitting, we will first try classification models with low complexities, such as naïve Bayes, softmax, and support vector machines (SVM) to establish a baseline, and then move to more complex models, such as decision trees, random forests, and neural networks [76–81]. The second approach (regression-based) will directly predict prevalence and malaria transmission intensity based on the predictive variables using Gradient Boosting (GB) linear and logistic regression (LogitBoost) or an ensemble of regression trees [82]. Each method has pros and cons. The classification-based approach is easier to interpret and less prone to overfitting, but the cluster assignment may not be clear-cut. On the other hand, although it does not require cluster assignment, the regression-based approach must model prevalence and vector abundance separately and is thus more prone to overfitting with nonlinear models [83].

The data from all study clusters (36 initially) will be split into a training set and a validation set, with model parameters trained on the training set and performance evaluated on the validation set. Tenfold cross-validation will be performed to maximally utilize the data [84, 85]. For the classification task, we will determine each model’s sensitivity and specificity and use the auROC (area under ROC curve) to measure model performance. For the regression task, we will use the mean-square loss to measure model performance. Trained models will be further tested and refined on data from 36 different clusters collected in year 2 and future years.

The goal is to stratify all clusters into 3 blocks during the initial intervention stage: low transmission, intermediate transmission, and high transmission.

### Trial design and outcome measures

#### Initial cluster randomization

**Fig. 2:**
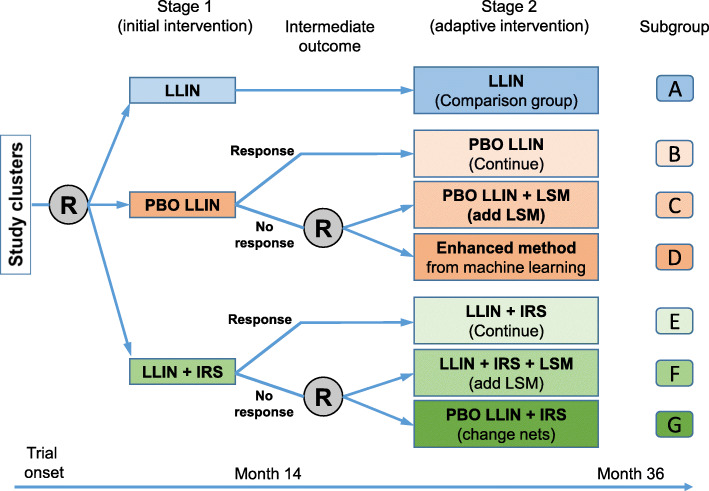
Sequential multiple assignment randomized trial (SMART) study for developing adaptive malaria intervention strategy in Kenya. R, randomization; LLIN, long-lasting insecticidal net; PBO LLIN, piperonyl butoxide-treated LLIN; IRS, indoor residual spraying; LSM, larval source management

The SMART trial design includes two intervention stages (Fig. 2 and Table 1). Based on pre-intervention malaria risk analysis, study clusters will be stratified into three blocks with different risk levels as described above. In each block, clusters will be randomly assigned with equal probability to one of the three interventions in stage 1, i.e., LLIN, PBO LLIN, and LLIN + IRS (Fig. 2). Cluster randomization will be done using computer-generated random numbers. Ideally, each block will include 12 clusters and each of the three interventions will be assigned to four clusters in each block (Fig. 1).

#### Stage 1 intervention

Stage 1 (initial intervention) will be 15 months long, including a 3-month field implementation of interventions and 12 months of follow-up on the interventions’ impact. The main purpose of stage 1 is to determine the effect of the individual interventions on malaria incidence and to identify clusters not responding to these interventions. At the beginning of stage 1, all study clusters will be randomized with equal probability to the three interventions, as shown in Fig. 2, by a block randomization method. At the end of the 12-month follow-up, clusters will be assessed for “response” versus “non-response” to PBO Nets or LLIN + IRS. Response and non-response are defined based on the change in clinical malaria incidence when using PBO Nets/LLIN + IRS compared to LLIN (Fig. 2). If a cluster demonstrates a reduction in malaria incidence that is (1) statistically significant and (2) greater than the pre-defined threshold value set by the Ministry of Health (MoH) based on cost-effectiveness, then this cluster is considered a responder; otherwise, the cluster is considered a non-responder. It is important to use the LLIN group (group A in Fig. 1) as the comparison group when determining response/non-response, because malaria incidence varies between seasons and years due to seasonal or inter-annual climatic variability. In addition, LLIN is the government-implemented first-line routine intervention.

#### Stage 2 intervention

The stage 2 intervention will be 21 months long, including a 3-month intervention implementation and 18 months of follow-up on the interventions’ impact. The main objective of stage 2 is to identify the optimal adaptive intervention in clusters not responding to the stage 1 intervention, i.e., the non-response clusters at the end of stage 1 will receive a modified intervention to improve the outcome (reduction of malaria incidence) (Fig. 2). In particular, non-responders to stage 1 PBO LLIN treatment will be randomized with equal probability to either PBO LLIN + LSM (larval source management) or an intervention determined by an enhanced reinforcement learning method. Similarly, non-responders to stage 1 LLIN + IRS treatment will be randomized with equal probability to either LLIN + IRS + LSM or PBO LLIN + IRS. Therefore, this design has 4 embedded adaptive interventions (Table 2).

**Table 2:** Four embedded adaptive interventions in the proposed SMART trial study

Embedded adaptive intervention	First-stage intervention	Status at end of first-stage treatment	Second-stage intervention option	Subgroup in the design
#1	PBO LLIN	Responder	Continue PBO LLIN	B + C
		Non-responder	PBO LLIN + LSM	
#2	PBO LLIN	Responder	Continue PBO LLIN	B + D
		Non-responder	Enhanced machine learning method	
#3	LLIN + IRS	Responder	Continue IRS	E + F
		Non-responder	LLIN + IRS + LSM	
#4	LLIN + IRS	Responder	Continue IRS	E + G
		Non-responder	PBO LLIN + IRS	

#### Enhanced machine learning method

A reinforcement learning-based method will be developed and deployed to adaptively assign treatments to different study clusters based on stage 1 intervention results and historical results. Assume *K* treatment options are available and each treatment’s outcome (*a*) is stochastic, depending not only on treatment but also on contextual cluster features (*s*) such as current malaria prevalence, incidence rate, and the socioeconomic, entomological, and genetic features described earlier. We will use a specific reinforcement learning algorithm, *Q*-learning [86], to design a function, *Q*(*s*, *a*), which calculates the expected reward (e.g., reduction of malaria incidence) if treatment *a* is applied to the study group with feature vector *s*. The *Q*-function measures each treatment method’s effectiveness conditioned on specific clusters and will be learned based on data collected from existing and ongoing trials. We will use linear functions, decision trees, or neural networks to model the *Q*-function [81, 87]. To avoid overfitting, we will start with simple linear models and progress toward more complex models such as decision trees and neural nets when more data become available. The enhanced method will automatically assign a treatment to each group based on the estimated *Q*-function. To achieve a balance between exploration and exploitation, we will assign treatments stochastically according to a probability vector calculated from the *Q*-function. A number of algorithms are possible, including **ε**-greedy, Boltzmann exploration, upper confidence bounds (UCB), and reinforcement comparison [88]. We will first test these algorithms in simulation studies and choose the best one for implementation in real trials.

#### Sample size justification

The primary aim of this study is to determine which initial intervention, PBO LLIN or LLIN + IRS, is more effective in reducing malaria incidence after 36 months. The second aim is to estimate the mean outcomes of the 4 embedded adaptive interventions from months 16 to 36 and to identify the most effective intervention. Sample size calculation is based on the primary aim, using the primary outcome (clinical malaria incidence) and between-group comparison of the average clinical malaria incidence at month 36. The proposed sample size (*n* = 36 clusters) will detect a 10% reduction in clinical malaria incidence with 89.2% power using the current incidence rate in the study sites (302 clinic cases per 1000 person-years) and a 2-tailed *α* = 0.05 with a human population size of 500 per cluster (Table 3). If malaria incidence is 30% lower than the current value, the design will still detect a 20% incidence reduction with 97% power (Table 3). This calculation assumes a 1:1 response/non-response ratio, a fixed cluster size of 500 participants, and an intra-cluster correlation coefficient of 0.05 [89]. This sample size is likely powered to detect smaller changes in incidence reduction, as previous studies in Kenya and Ghana indicated intra-cluster correlation coefficients of < 0.02 [90].

**Table 3:** Power calculation (%) for proposed cluster-randomized SMART trial. Shown are degrees of power to detect four levels of incidence reduction under three incidence scenarios

Annual incidence rate (cases/1000 population)	Reduction in malaria incidence			
	40%	30%	20%	10%
Observed in the site: 302	> 99.9	> 99.9	> 99.9	89.2
30% lower value: 211	> 99.9	99.8	97.0	59.0
30% higher value: 393	> 99.9	> 99.9	> 99.9	97.0

### Active malaria case detection and asymptomatic infection survey

Clinical malaria will be identified through active case detection. A cohort of 150 households, which includes about 500 residents based on our previous surveys in the same area (household size of 3.35 or 14,824/4420 persons/household), will be selected randomly from each cluster, and all residents in the selected households will be recruited upon signing consent/assent forms for participation. Written informed consent/assent (for minors under age of 18) for study participation will be obtained from all consenting heads of households and each individual who is willing to participate in the study. Inclusion criteria are provision of informed consent/assent and no reported chronic or acute illness except malaria. Exclusion criteria are individuals who are unwilling to participate or infants under the age of 6 months. Participants will be visited every 2 weeks and screened for clinical malaria. A clinical malaria case is defined as an individual with fever (axillary temperature of 37.5 °C or higher) and other related symptoms such as chills, severe malaise, headache, or vomiting at the time of examination or 1–2 days prior to the examination, in the presence of a *Plasmodium*-positive blood smear.

During each visit, a project field team consisting of a lead laboratory technician, a community health volunteer, and a field assistant will talk to the matriarch, who has information on the health status of every resident in the household, to check if any resident in the household has experienced fever within the last 48 h or is suspected to have malaria. For all fever and suspected malaria cases, blood will be taken and thin and thick smears prepared on a labeled slide. Body temperature will be taken with a digital thermometer, and the symptoms and signs of the illness will be recorded on a case report form (CRF). Clinical cases will be referred to the nearest government-run hospital or health center for free treatment. Each participant will be assigned a unique identification number corresponding to their household and cluster. Each participant will also receive an identity card, which can be used to obtain free malaria treatment at the hospital or health center whenever they have a fever or believe they have malaria.

Asymptomatic malaria infection is important for malaria control. To determine the impacts of interventions on malaria infection prevalence, we will collect finger-prick blood samples as described in baseline surveys. Study subjects are selected from the same households as the active case detections. Finger-prick blood samples will be collected on blood slides and on filter papers for PCR analysis, i.e., to determine infection status. The survey will be done twice a year, once in the rainy season and another time during dry season. Age and sex of participants will be recorded.

We masked field staff, who will conduct the active case detections and collect blood samples and mosquito samples in the cross-sectional surveys, to the study groups the clusters were assigned to. However, it is not possible to mask either the investigators or the participants to the treatment allocation of indoor residual spraying.

### Informed consent, ethical clearance, and conflict of interest statement

Ethical clearance has been obtained from the Ethical Review Committee of Maseno University, Kenya (MSU/DRPI/MUERC/00778/19), and the Institutional Review Board (IRB) of the University of California, Irvine, USA (HS# 2017-3512). Written consent will be obtained from all participants. Written assent for children (< 18 years of age) will be obtained from the participants and their parents or guardians. Inclusion criteria are as follows: provision of informed consent (assent for children) and no reported chronic or acute illness other than malaria. Exclusion criteria are as follows: unwillingness to participate in the study or reported chronic or acute illness other than malaria. Permission to use microbial larvicides for malaria vector control has been obtained from the Pest Control Products Board of Kenya. All investigative team members in the USA and Kenya have no financial conflict of interest with the larvicide manufacturer, Central Life Sciences.

### Malaria vector population monitoring

We will conduct malaria vector population surveillance on a seasonal basis continuously until at least 12 months after the stage 2 intervention (Fig. 2). We will monitor both indoor- and outdoor-biting mosquito abundance using non-baited CDC light traps. We will set up two traps within each sampling compound: one inside the living room and the other outside the house 5 m away. We will conduct a total of 40 trap-nights (20 indoor and 20 outdoor) of vector sampling per cluster per season. Species of collected mosquitoes will be identified, and blood-feeding status will be recorded. We will test for *P. falciparum* sporozoite infection and blood meal source using an enzyme-linked immunosorbent assay (ELISA) on all specimens [55]. For each house where the vector population is sampled, we will record the number of sleeping persons at the house on the day of the vector survey. We will calculate the sporozoite rate and EIR for each cluster. The EIR will be calculated as (number of *Anopheles* per person) × (average number of persons bitten by one *Anopheles* in 1 day) × (sporozoite rate) and standardized to a seasonal basis. We will calculate indoor and outdoor transmission intensities separately, assuming that all mosquitoes collected from a compound had their blood meal from the same household. We will calculate the EIR for each cluster, if possible, or for each block. In addition to population monitoring, we will also conduct insecticide resistance monitoring twice a year.

### Cost-effectiveness analysis

The cost-effectiveness analysis will assess the economic costs of each phase of the PBO LLIN, IRS, or LSM from both a provider and a societal perspective, using standard economic evaluation methodologies [91]. Economic costs will be estimated for all areas of resource use, regardless of whether they incur a financial expense [92]. For example, if IRS or larvicides have been donated or community volunteers are helping with the intervention free of charge, an economic cost will attach a market value to these resources. Economic costs will include initial setup investment (e.g., capital for vehicles used in transporting the insecticides; GPS units for house or habitat mapping; pumps, storage space, and equipment; and traps for mosquito surveillance), running costs (e.g., insecticides, salaries for field application staff, staff training, protective clothes, gloves, fuel costs, and vehicle insurance), and costs of program management and quality control (e.g., material procurement, project coordinator, and quality controller). The initial setup cost will be annuitized over each asset’s useful lifetime. This process reflects the value-in-use of a capital asset, not the cost when the item was purchased. We will record the number of person-hours used per month and the quantity of insecticides used to accomplish the intervention during the above experimental activities. Cost data on equipment and supplies will be obtained from local markets in Kenya and Ethiopia, labor costs from the MoH of Kenya and Ethiopia, and insecticide costs from the manufacturers. Costs will be measured in the currency in which the resources are paid, then converted to US dollars. This costing method includes costs involved in field intervention, quality control, and program evaluation. Costs associated with academic research will be excluded. Incremental cost-effectiveness ratios will be based on the primary endpoint (i.e., the economic cost per clinical malaria case prevented). One-way and multi-way sensitivity analysis will examine the cost-effectiveness implications of potential changes in labor cost, insecticide price, and insecticide application frequency and efficacy [93]. Costs and effects will be presented in both discounted and undiscounted form. Cost-effectiveness results will be compared among the two intervention groups and the control group, and among the four embedded adaptive interventions.

### Other related surveys

Similar bioassays or questionnaire surveys have been done by our group in different sites in western Kenya; details have been described in our previous studies [8, 94, 95]. Briefly, mosquito insecticide resistance surveys will be done twice a year to monitor if mosquitoes develop resistance to the newly introduced IRS and PBO LLIN interventions. Mosquito resistance to insecticides will be determined using standard WHO tube bioassays [8]. Insecticide decay will be tested using standard WHO cone test [94]. This will be done twice a year. The test will include both LLINs and IRS wall materials; details of the tests have been described in our previous study [94]. Questionnaire surveys on bed net ownership, net usage, and net physical integrity will be done once a year [95].

### Data analysis

Differences in clinical malaria incidence and vector abundance between the treatment and control groups will be analyzed using Poisson multivariate regression models with intervention, malaria risk, cluster, and calendar time as covariates, using a generalized estimating equation (GEE) approach with population at each cluster as an offset [96]. GEE is necessary because incidence will be modeled monthly as a longitudinal measure using grouped data. Intervention will be a time-varying covariate, since the treatment in a cluster may be adapted and readapted depending on the response to the previous treatment. The odds ratio and 95% confidence interval for clinical malaria rates between the treatment groups and the control group will be calculated. For the second aim analysis (estimation of outcomes for the 4 embedded adaptive interventions), weighted and replicated generalized estimating equations will be used to estimate the mean clinical malaria incidence rates among the 4 embedded adaptive interventions and to compare the slopes at each stage for each adaptive intervention. Here, weighting is necessary to account for the potential over- or underrepresentation of some groups (e.g., non-responders would have a 1/4 chance of following their assigned treatment sequence, whereas responders would have a 1/2 chance of following their assigned treatment sequence) [97, 98].

We will identify a final intervention strategy after all cost and effectiveness data are collected. The aim of this strategy will be to recommend an optimal intervention method given the local conditions at each site. For this purpose, we will adopt the *Q*-learning framework from the previous section on enhanced machine learning methods [99]. We will train a neural network to predict each method’s utility, accounting for both effectiveness (reduction in malaria incidence rate) and cost based on the conditions at each site, such as malaria risk and socioeconomic, entomological, and genetic features. All data collected throughout this research will be used to train the final model. To reduce model complexity, we will consider clustering sites into a small number of classes and then training a model to predict the efficacy of each method conditioned on each class. Once the *Q*-function is learned, we will identify the optimal intervention by choosing the intervention method that maximizes the learned *Q*-function.

## Discussion

The continuing high malaria burden in many areas of Africa calls for improving the effectiveness of malaria intervention tools [1]. This will require optimizing current first-line interventions and integrating newly approved tools into control programs [100]. Due to the heterogeneity and dynamic changes in malaria risk and vector ecology among epidemiological settings, interventions that work in one setting may not work well in others. This trial study aims to develop adaptive intervention strategies tailored toward local malaria risks and vector ecologies based on the available set of interventions and cost factors at a point in time. Such an adaptive strategy is expected to enhance the efficiency of malaria interventions. If adaptive interventions built from modern data analytic methods work well, they could provide new malaria vector control strategies in Africa and other endemic areas where malaria incidence is high or has rebounded since the implementation of currently used intervention tools.

Randomized controlled trial (RCT) design has been used to evaluate malaria control interventions [28, 101–103]. The SMART design represents a significant departure from standard RCT design, in which the intervention method is fixed a priori. In contrast, in the SMART design, the intervention method is adaptive, i.e., data from the early response to an intervention and predicted future risks are used to determine the next intervention method for those enrolled in the trial. Adaptive intervention is necessary for several reasons. First, given the many available and emerging malaria vector control tools, finding the best intervention or combination of interventions is not easy. It would be very difficult if not impossible to design an RCT due to the potentially huge sample size needed. Second, due to the heterogeneity and dynamic nature of transmission, interventions must adapt and readapt to the changing epidemiology. Third, standard RCT does not allow interventions to be adjusted during the trial, whereas adaptive intervention allows for changes, making it easier to find the best-suited intervention. Therefore, results from the adaptive intervention provide evidence for effective interventions that are closely tailored to local malaria risks, vector ecologies, and intervention logistics. To our knowledge, this is the first cluster-randomized SMART for malaria.

Recent developments in predictive data analytic techniques and machine learning provide novel tools to assist in identifying mosquito larval habitats and assessing malaria risks [101, 102]. Machine learning, such as reinforcement learning, has been used to identify optimal intervention strategies in clinical treatment studies [80, 86–88]. Reinforcement learning techniques are being used increasingly in the area of personalized medicine [103, 104] but have not previously been used for malaria. We will use *Q*-learning to identify larval habitats and to find the optimal intervention suited to local malaria epidemiological settings. The trial will include larviciding using long-lasting larvicides and IRS with new insecticides. The combination of satellite image analysis and machine learning will aid significantly in locating mosquito breeding sites and implementing IRS.

### Trial status

This trial is underway. Pre-intervention epidemiological and entomological surveillances were started in July 2019. Cluster selection, stratification, and randomization have been done. Demographic surveillance has been conducted once, in July 2019. Cohort active case surveillance was initiated in October 2019 and officially started in March 2020. The stage 1 intervention started recruiting in late February 2020, and recruitment will continue until March 2021 as anticipated. Post-intervention follow-ups started in March 2020 till February 2022. The trial was registered with US National Institutes of Health at ClinicalTrials.gov, study ID NCT04182126. Registered on 26 November 2019 and last updated on 3 March 2020. The manuscript was prepared when the trial protocol was registered; trial protocol was submitted for publication when recruitment of study participant was ongoing.

## Data Availability

Not applicable.

## Abbreviations

Bs: *Bacillus sphaerius*
Bti: *Bacillus thuringiensis israelensis*
CDC: US Centers for Disease Control and Prevention
CRF: Case report form
EIR: Entomological inoculation rate
ELISA: Enzyme-linked immunosorbent assay
EPA: US Environmental Protection Agency
GEE: Generalized estimating equation
GIS: Geographic information system
GPS: Global positioning system
IRS: Indoor residual spraying
LLIN: Long-lasting insecticidal net
LSM: Larval source management
MoH: Ministry of Health
PBO: Piperonyl butoxide
RCT: Randomized controlled trial
SMART: Sequential multiple assignment randomized trial
